# Serum amyloid alpha 1-2 are not required for liver inflammation in the 4T1 murine breast cancer model

**DOI:** 10.3389/fimmu.2023.1097788

**Published:** 2023-02-03

**Authors:** Chenfeng He, Riyo Konishi, Ayano Harata, Yuki Nakamura, Rin Mizuno, Mayuko Yoda, Masakazu Toi, Kosuke Kawaguchi, Shinpei Kawaoka

**Affiliations:** ^1^ Inter-Organ Communication Research Team, Institute for Life and Medical Sciences, Kyoto University, Kyoto, Japan; ^2^ Department of Breast Surgery, Kyoto University Graduate School of Medicine, Kyoto, Japan; ^3^ Department of Integrative Bioanalytics, Institute of Development, Aging and Cancer (IDAC), Tohoku University, Sendai, Japan

**Keywords:** cancer-induced systemic inflammation, acute phase response, serum amyloid alpha, 4T1 breast cancer, neutrophils, bone marrow, liver

## Abstract

Cancers induce the production of acute phase proteins such as serum amyloid alpha (SAA) in the liver and cause inflammation in various host organs. Despite the well-known coincidence of acute phase response and inflammation, the direct roles of SAA proteins in inflammation in the cancer context remains incompletely characterized, particularly *in vivo*. Here, we investigate the *in vivo* significance of SAA proteins in liver inflammation in the 4T1 murine breast cancer model. 4T1 cancers elevate the expression of SAA1 and SAA2, the two major murine acute phase proteins in the liver. The elevation of *Saa1-2* correlates with the up-regulation of immune cell-related genes including neutrophil markers. To examine this correlation in detail, we generate mice that lack *Saa1-2* and investigate immune-cell phenotypes. RNA-seq experiments reveal that deletion of *Saa1-2* does not strongly affect 4T1-induced activation of immune cell-related genes in the liver. Flow cytometry experiments demonstrate the dispensable roles of SAA1-2 in cancer-dependent neutrophil infiltration to the liver. Consistently, 4T1-induced gene expression changes in bone marrow do not require *Saa1-2*. This study clarifies the negligible contribution of SAA1-2 proteins in liver inflammation in the 4T1 breast cancer model.

## Introduction

Inflammation in host organs is a major phenomenon caused by advanced, incurable solid cancers (1–4). Advanced solid cancers induce the proliferation of particular immune cell types, expression of inflammatory cytokines, and migration of immune cells to particular organs such as the liver. These abnormalities are generally associated with a worse prognosis for cancer patients (5–8). Understanding how cancer cells modulate the host immune system is thus an important area of research for developing therapeutics that could mitigate cancer’s adverse effects on the host.

Acute phase response, traditionally known as the increased plasma concentration of the liver-derived secretory proteins (i.e., acute phase proteins) upon exposure to stimuli, is observed in various animal cancer models and cancer patients (3, 6, 9–12). Serum amyloid alpha (SAA) is a representative acute phase protein (9). In the presence of stimuli such as infection and advanced cancers, hepatocytes produce large amounts of SAA proteins (9, 13, 14). The plasma concentration of SAA proteins consequently elevates, modulating the immune system in various manners. In this regard, acute phase proteins are considered liver-derived amplifiers of systemic immune response to stimuli.

Previous studies have suggested that SAA proteins can modulate the activity of particular immune cell types (9). For example, human SAA proteins promote the cytokine release of neutrophils (9, 15). Macrophages are also a target of SAA (16–18). SAA proteins were also reported to induce muscle atrophy *via* Toll-like receptors (19). Notably, these findings are often based on *in vitro* and ex vivo experiments using exogenous SAA proteins (9), whereas less is known about the contribution and significance of endogenous SAA proteins to systemic inflammation *in vivo*. This insufficient understanding is at least in part due to the relatively small number of studies using knockout of *Saa* genes *in vivo*.

In the present study, we explore the effects of genetic deletion of SAA1 and SAA2 (hereafter referred to as SAA1-2), the two major SAA proteins in mice, on liver inflammation caused by 4T1 breast cancers. We find that 4T1 breast cancers activate the host immune system even in the absence of SAA1-2 proteins, suggesting the dispensable roles of SAA1-2 in cancer-induced liver inflammation in this particular breast cancer model.

## Results

### 4T1 breast cancers elevate SAA1-2 in the liver

**Figure 1:**
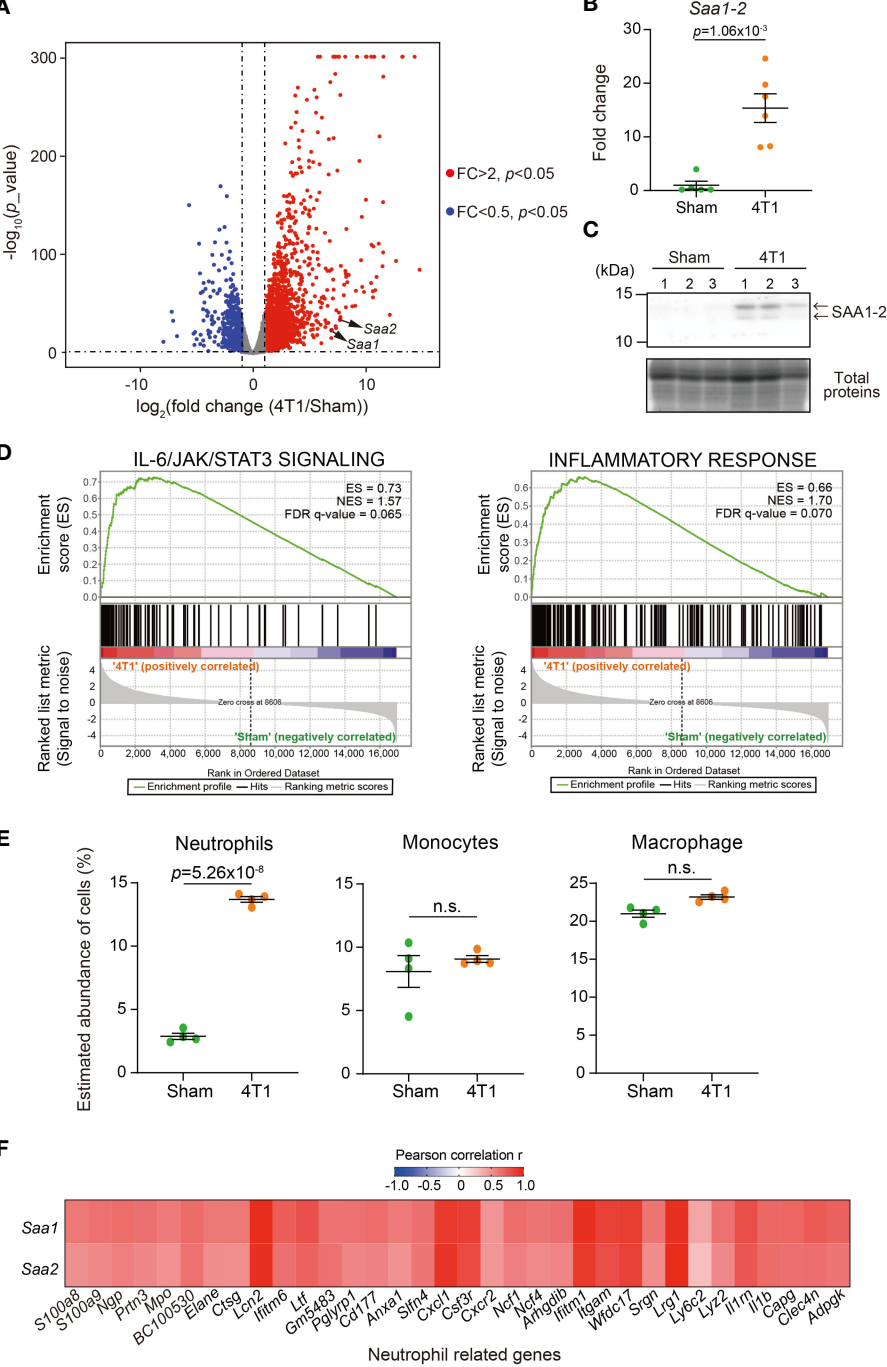
4T1 breast cancers elevate SAA1-2 in the liver **(A)** RNA-seq experiments for the livers of sham-operated mice and 4T1-bearing mice in WT (14 days after 4T1 transplantation). A volcano plot (log_2_ (fold change (4T1/Sham)) versus –log_10_ (*p* value)) of WT is shown. Genes showing more than 2-fold change with *p* < 0.05 are highlighted. *n* = 4. **(B)** qPCR analysis of *Saa1-2* in the livers of sham and 4T1-bearing mice. Averaged fold change data normalized to the sham group are presented as the mean ± SEM. The *p* value is shown (unpaired two-tailed Student’s *t*-test). *n* = 5 for sham-operated mice and *n* = 6 for 4T1-bearing mice. **(C)** Western blot analysis for SAA1-2 in the livers of sham and 4T1-bearing mice. *n* = 3. **(D)** GSEA plots that evaluate hepatic gene expression changes in “IL-6/JAK/STAT3 signaling” and “Inflammatory response” upon 4T1 transplantation. FDR *q* value, enrichment score (ES), and normalized enrichment score (NES) are shown. **(E)** Dot plots showing the estimated abundance of the indicated immune cell types in the liver of sham and 4T1-bearing mice. The scores are calculated using the RNA-seq datasets in Figure 1A and ImmuCellAI-mouse. Data are mean ± SEM. The *p* value is shown (unpaired two-tailed Student’s *t*-test). n.s., not significant. **(F)** Heatmap representation of the correlations between *Saa1-2* and representative neutrophil-related genes at mRNA level using the RNA-seq dataset **(A)**. *n* = 4. See also **Figure S1B**.

We previously demonstrated that transplantation of 4T1 breast cancer cells to BALB/c female mice strongly increased the expression of *Saa1-2* mRNAs in the liver (14). We re-analyzed the RNA-seq datasets we recently published (20) to confirm that *Saa1-2* mRNAs were induced in the livers of 4T1 breast cancer-bearing mice (**Figure 1A**). We further validated this observation using reverse transcription quantitative PCR (RT-qPCR) detecting both *Saa1* and *Saa2* whose nucleotide sequences are 95% identical (**Figure 1B** and **Figure S1A** and **Table S1**). We also found that 4T1 breast cancers elevated hepatic SAA1-2 at the protein level (**Figure 1C**). Together, we concluded that transplantation of 4T1 breast cancers enhanced acute phase response in the liver, which is in line with other cancer models (13).

To address whether the increased expression of SAA1-2 affects inflammation, we characterized our liver transcriptome data using gene set enrichment analysis (GSEA) (**Figure 1D**) (21, 22). Our analysis revealed that 4T1 breast cancer triggered various inflammatory responses in the livers as exemplified by the activation of the interleukin-6 (IL-6) signaling (23). This was in line with the previous observations that *Saa1-2* genes are under the control of the IL-6 signaling (13) and that solid cancers instigate the IL-6 signaling in the liver (3, 23). We then used ImmuCellAI-mouse to deduce the infiltration of various immune cells into the liver (24, 25). Using this method, we found that neutrophils migrated into the liver upon cancer transplantation (**Figure 1E**), as previously reported (13, 14). Furthermore, we noted the close correlations at the mRNA level between *Saa1-2* and other neutrophil-related genes such as *Lcn2* (**Figure 1F** and **Figure S1B**). Together with the known biochemical roles of SAA1-2 (9), these results led to a hypothesis that SAA1-2 proteins play some roles in immune cell activation in the presence of 4T1 breast cancers.

### Generation of mice completely lacking SAA1-2

**Figure 2:**
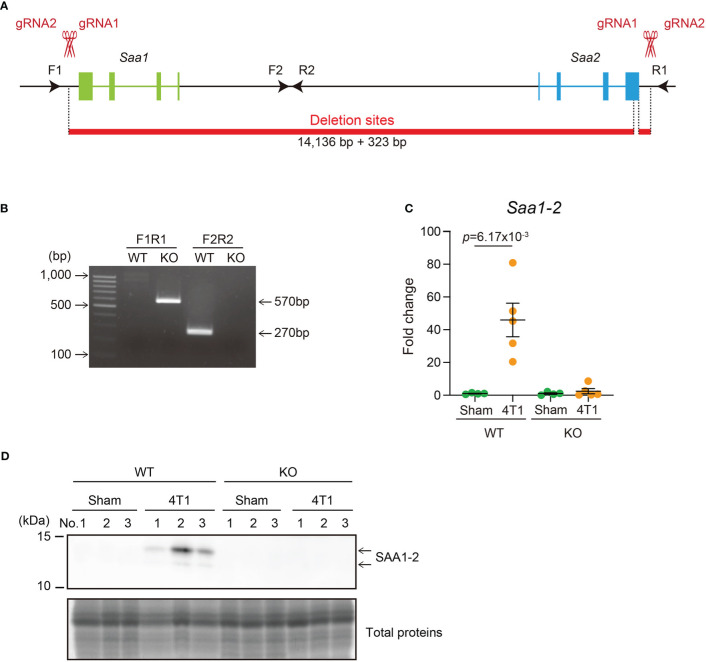
Generation of mice completely lacking SAA1-2 **(A)** Schematic representation of *Saa1-2* deletion using the CRISPR-Cas9 system. The gRNA-targeted sites and the primers used for genotyping experiments in **(B)** are indicated. The deleted regions are indicated in red. **(B)** A representative image of genomic PCR against the *Saa1-2* locus. **(C)** qPCR analysis of *Saa1-2* in the livers of sham and 4T1-bearing mice in WT and *Saa1-2* KO. Averaged fold change data normalized to the sham group in each genotype are presented as the mean ± SEM. The *p* value is shown (unpaired two-tailed Student’s *t*-test). *n* = 4 for sham-operated mice and *n* = 5 for the 4T1-bearing mice. **(D)** Western blot analysis for SAA1-2 in the livers of sham and 4T1-bearing mice in WT and *Saa1-2* KO. *n* = 3. See also **Figure S2**.

To uncover the roles of SAA1-2 in inflammation *in vivo* in this model, we generated mice completely lacking *Saa1* and *Saa2*. These two genes are located closely in the murine genome (**Figure 2A**), having redundant sequences and molecular functions (**Figure S1A**) (9). We thus decided to delete the entire region encoding *Saa1* and *Saa2* genes by designing gRNAs on the right and left sides of this genomic locus (**Figure 2A**) (26). As a result, we succeeded in deleting both *Saa1* and *Saa2* as determined by genomic PCR (**Figures 2A, B**). Transplantation of 4T1 breast cancer cells to *Saa1-2* knockout mice no longer increased the expression of *Saa1-2* mRNAs (**Figure 2C**) and proteins (**Figure 2D**) in the liver. Western blot experiments demonstrated that two bands detected in lysates prepared from the livers of 4T1-bearing mice disappeared in *Saa1-2* knockout mice (**Figure 1C** and **Figure 2D**). Adding recombinant SAA1 protein that lacks the signal peptide as a control, we reasoned that the lower band corresponds to the cleaved SAA proteins (**Figure S2**). Taken together, we established mice where we completely canceled cancer-dependent increase in SAA1-2 proteins in the liver, allowing us to investigate the *in vivo* significance of these proteins in cancer-induced liver inflammation.

### Deletion of *Saa1-2* does not have strong impacts on liver transcriptome

**Figure 3:**
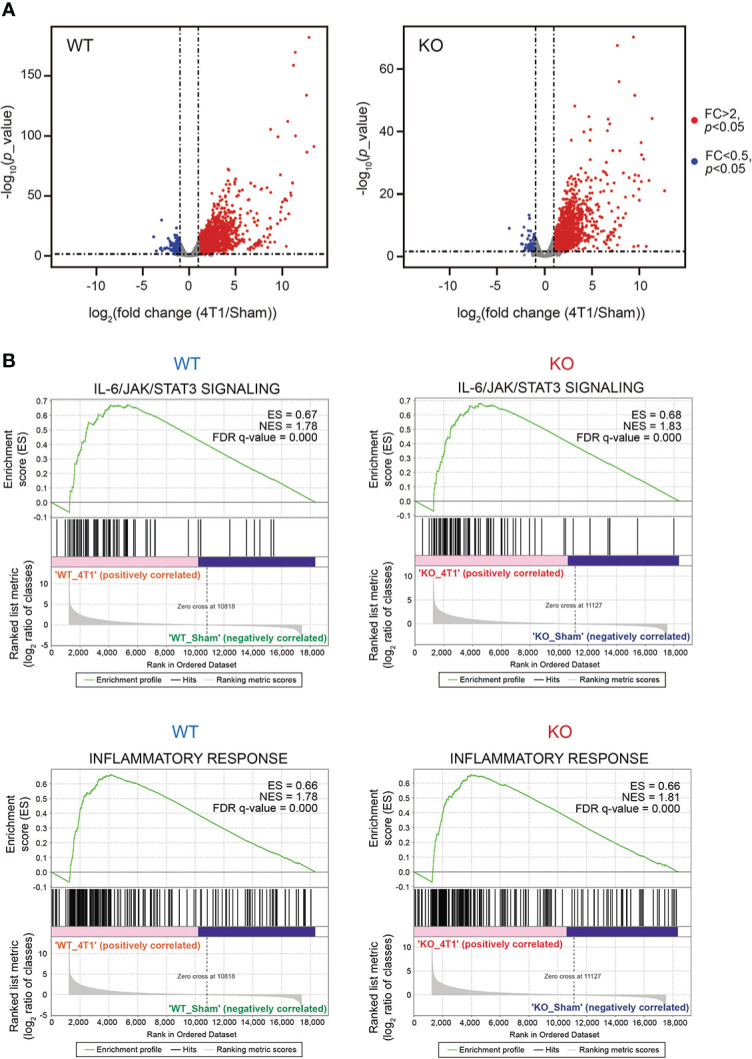
Deletion of *Saa1-2* does not have strong impacts on liver transcriptome **(A)** RNA-seq experiments for the livers of sham-operated mice and 4T1-bearing mice in WT and *Saa1-2* KO (14 days after 4T1 transplantation). Volcano plots (log_2_ (fold change (4T1/Sham)) versus –log_10_ (*p* value)) of WT (left) and *Saa1-2* KO (right) are shown. Genes showing more than 2-fold change with *p* < 0.05 are highlighted in red. *n* = 2 for the sham groups and *n* = 3 for 4T1-bearing groups. See also **Figure S3** for qPCR analyses using the different cohorts of 4T1 transplantation experiments. **(B)** GSEA plots that evaluate hepatic gene expression changes in “IL-6/JAK/STAT3 signaling” and “Inflammatory response” upon 4T1 transplantation in WT and *Saa1-2* KO. FDR *q* value, enrichment score (ES), and normalized enrichment score (NES) are shown.

To thoroughly analyze the effects of *Saa1-2* knockout on the liver transcriptome, we performed RNA-seq analyses against the livers of WT and *Saa1-2* knockout mice (**Figure 3A** and **Table S2**). We found that 4T1 breast cancer transplantation similarly affected liver transcriptome regardless of the presence or absence of *Saa1-2* genes, as evidenced by the volcano plots shown in **Figure 3A** and **Figure S3A, B**. GSEA demonstrated that 4T1 breast cancers could still activate the IL-6 signaling and inflammatory response in the absence of *Saa1-2* genes (**Figure 3B**), suggesting a subtle or negligible contribution of *Saa1-2* to the liver transcriptome in our experimental settings. In addition, we wanted to confirm these observations using different cohorts of 4T1 transplantation experiments. For this purpose, we quantified the mRNA expression of various immune cell marker genes in the liver, finding that *Saa1-2* KO did not have a significant impact on cancer-dependent up-regulation of representative immune-related genes in the liver (**Figure S3C**). These results suggested that 4T1 breast cancer cells do not require *Saa1-2* to increase the expression of immune-related genes in the liver in our experimental settings.

### SAA1-2 are dispensable for 4T1-induced infiltration of immune cells to the liver

A previous study reported the significant contribution of SAA proteins in recruiting innate immune cells including neutrophils to the liver in the pancreatic cancer-bearing condition (13). This prompted us to further investigate the roles of SAA1-2 in immune cell recruitment to the liver in the 4T1 breast cancer model. To this end, we estimated the proportions of several immune cell types in the livers of cancer-bearing mice, comparing them between WT and *Saa1-2* KO.

**Figure 4:**
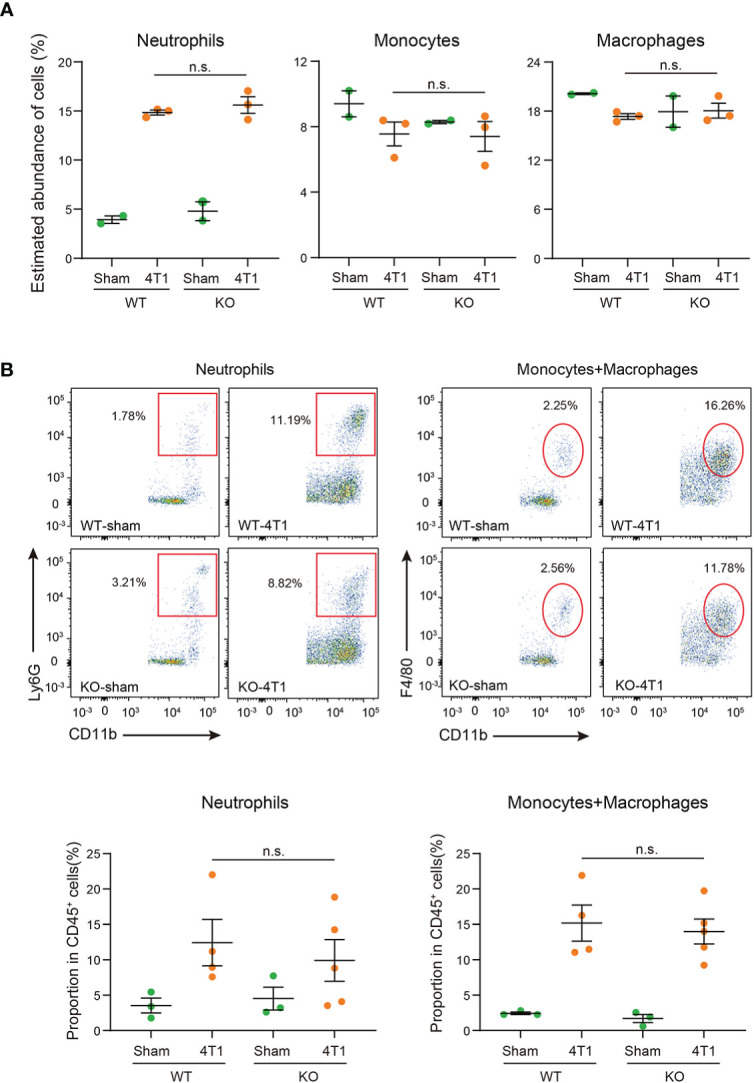
SAA1-2 are dispensable for 4T1-induced infiltration of immune cells to the liver **(A)** Dot plots showing the estimated abundance of the indicated immune cell types in the liver of sham and 4T1-bearing mice. The scores are calculated using the RNA-seq dataset in Figure 3A and ImmuCellAI-mouse. Data are presented as the mean ± SEM. n.s., not significant, unpaired two-tailed Student’s *t*-test. **(B)** Flow cytometric analysis of Ly6G^+^CD11b^+^ neutrophils and F4/80^+^CD11b^+^ monocytes and macrophages in the livers of sham and 4T1-bearing mice in WT and *Saa1-2* KO. Representative plots are shown. Data are mean ± SEM. The *p* value is shown (unpaired two-tailed Student’s *t*-test). n.s., not significant. *n* = 3 for the sham groups, *n* = 4 for 4T1-bearing WT mice, and *n* = 5 for 4T1-bearing *Saa1-2* KO mice. See also **Figure S4** for the gating strategies used in this study.

ImmuCellAI-mouse analyses confirmed that transplantation of 4T1 breast cancer cells increased the proportion of neutrophils within the liver (**Figure 4A** and **Table S3**). Of note, the proportions of neutrophils in the liver were comparable between WT and *Saa1-2* KO in the cancer-bearing condition, implying negligible roles of SAA1-2 in recruiting neutrophils in this model. We also investigated the proportions of neutrophils, monocytes, and macrophages using flow cytometry. We collected immune cells from the livers of sham and cancer-bearing animals, quantifying those immune cells using a set of specific antibodies (see figure legends and methods). As shown in **Figure 4B**, our data revealed that the proportions of neutrophils, monocytes, and macrophages were unaffected by *Saa1-2* KO. Collectively, although 4T1 breast cancer transplantation strongly induced SAA1-2 in the liver, these acute phase proteins do not appear to be essential for cancer-dependent immune cell recruitments into the liver in the 4T1 model (**Figures 1**-**4**).

### Dispensable roles of SAA1-2 in 4T1-induced transcriptomic changes in the bone marrow

Innate immune cells such as neutrophils are born and matured in the bone marrow (27). It is also known that cancers affect immune cell development (4). Given these, it was likely that the altered immune cell status in the liver was owing to changes in the bone marrow. We thus wanted to extend our experiments on the roles of *Saa1-2* in immune cell activation in the bone marrow.

**Figure 5:**
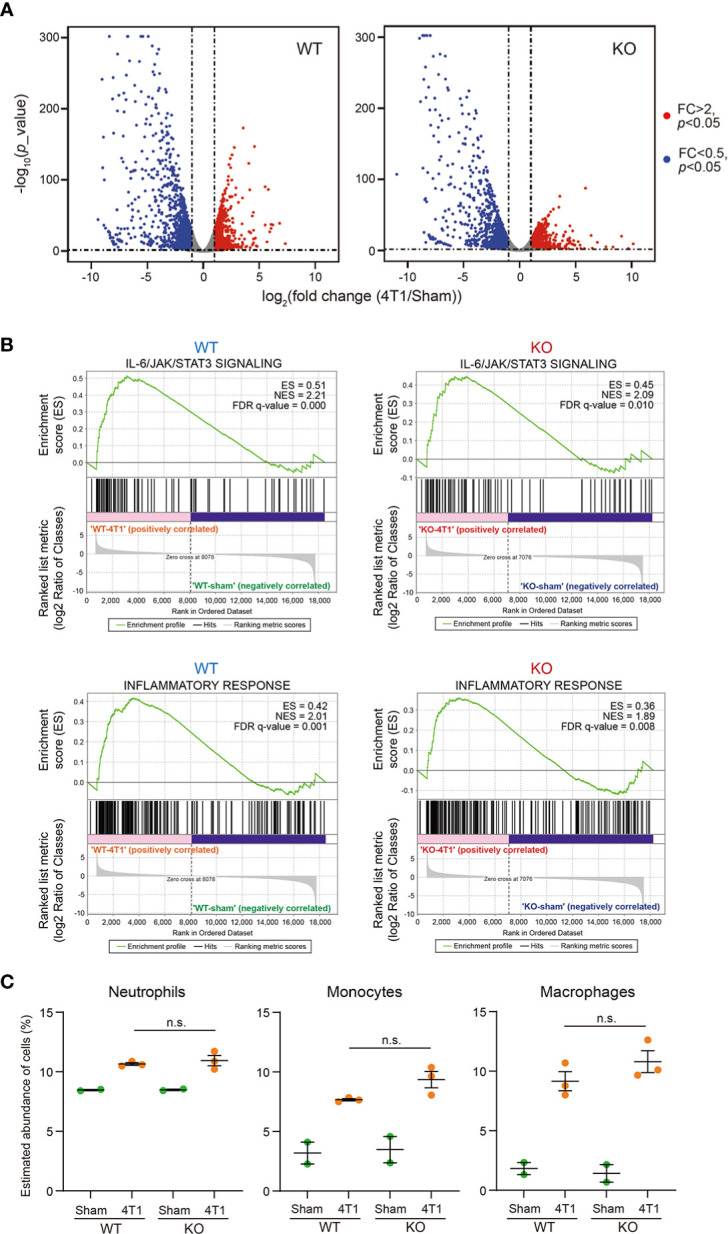
Dispensable roles of SAA1-2 in 4T1-induced transcriptomic changes in the bone marrow **(A)** RNA-seq experiments for the bone marrows of sham-operated mice and 4T1-bearing mice in WT and *Saa1-2* KO (14 days after 4T1 transplantation). Volcano plots (log_2_ (fold change (4T1/Sham)) versus –log_10_ (*p* value)) of WT (left) and *Saa1-2* KO (right) are shown. Genes showing more than 2-fold change with *p* < 0.05 are highlighted in red. *n* = 2 for the sham groups and *n* = 3 for 4T1-bearing groups. See also Figure S5 for qPCR analyses using the different cohorts of 4T1 transplantation experiments. **(B)** GSEA plots that evaluate gene expression changes in the bone marrow in “IL-6/JAK/STAT3 signaling” and “Inflammatory response” upon 4T1 transplantation in WT and *Saa1-2* KO. FDR *q* value, enrichment score (ES), and normalized enrichment score (NES) are shown. **(C)** Dot plots showing the estimated abundance of the indicated immune cell types in the bone marrows of sham and 4T1-bearing mice. The scores are calculated using the RNA-seq dataset in **(A)** and ImmuCellAI-mouse. Data are presented as the mean ± SEM. The *p* value is shown (unpaired two-tailed Student’s *t*-test). n.s., not significant, unpaired two-tailed Student’s *t*-test.

We performed RNA-seq experiments against immune cells collected from the bone marrow from WT and *Saa1-2* KO mice from which we obtained the liver transcriptome data (**Figure 3** and **Table S2**). We found that 4T1 transplantation affected gene expression in the bone marrow, resulting in many differentially expressed genes (**Figure 5A**, **Figure S5A**, and **Table S4**). According to GSEA, in WT, 4T1 transplantation activated the IL-6 signaling and inflammatory response (**Figure 5B**), which is in line with our liver data (**Figure 3B**). These results implied that inflammatory response observed in the liver is correlated with altered immune cell dynamics in the bone marrow. Moreover, ImmuCellAI-mouse analysis demonstrated that 4T1 transplantation altered the proportions of various cell types including neutrophils in the bone marrow, supporting a cancer-induced reprogramming of the host immune system (**Figure 5C** and **Table S3**) (4). Most importantly, none of these immune cell phenotypes in the bone marrow was strongly buffered by the deletion of *Saa1-2*. We validated these data using qPCR with the different cohorts of experiments (**Figure S5B**). These results together provided evidence that the effects of *Saa1-2* KO on the bone marrow transcriptome were negligible or minor if any, suggesting dispensable roles of SAA1-2 in 4T1-induced reprogramming of the host immune system. Thus, our results suggested that SAA1-2 proteins are not essential for liver inflammation observed in the 4T1 breast cancer model.

## Discussion

Advanced cancers reprogram the host immune system, thereby inducing systemic inflammation (1–6). Advanced cancer-induced inflammation in host organs is associated with the elevation in the levels of acute phase proteins both in murine cancer models and cancer patients, indicating the tight connection between inflammation and acute phase response in the cancer contexts (3, 6, 9–12). However, the causal relationship between these phenomena remains insufficiently addressed especially *in vivo*.

Using the 4T1 breast cancer model and *Saa1-2* KO mice, we evaluated the contribution of *Saa1-2* in 4T1-induced liver inflammation. As shown in **Figure 1**, the expression of *Saa1-2* showed significant correlations with immune cell gene expression and proportions of immune cells. In particular, the correlation between *Saa1-2* and innate immune cells including neutrophils appeared strong (**Figure 1**). This prompted us to generate mice completely lacking both *Saa1* and *Saa2* (**Figure 2**). Combining *Saa1-2* KO mice, RNA-seq, and flow cytometry, we found that, despite the strong correlation between SAA1-2 and various immune phenotypes, SAA1-2 proteins are not essential for the 4T1-induced immune cell alterations (**Figures 3**–**5**). Thus, 4T1 cancer cells can reprogram the host immune system independently of SAA1-2.

We do not exclude the possibility that our experimental settings had factors that mask the significance of SAA1-2. For example, the 4T1 breast cancer model induced the expression of *Saa3* (**Figure S6A**). SAA3 is a protein whose amino acid sequence is approximately 62% similar to those of SAA1 and SAA2 (**Figure S6B**). SAA3 plays important role in the pathogenesis related to T helper 17 (Th17) cells (28). Our data demonstrated that *Saa3* was still induced in both the liver and bone marrow of *Saa1-2* KO mice (**Figure S5B** and **S6A**), possibly compensating for the absence of *Saa1-2*. It is also possible that 4T1-derived cytokines are sufficient to induce and maintain liver inflammation (29). Given the massive number of transplanted 4T1 cancer cells in our experimental settings, the overwhelming amounts of cancer-derived cytokines might have concealed the significance of SAA1-2 proteins produced by the liver on inflammation. Our notion that experimental conditions might influence the roles of SAA1-2 does not contradict the previous report showing the critical role of SAA proteins in recruiting neutrophils to the liver in the presence of pancreatic cancers (13). In this regard, investigating SAA1-2 in other cancer models is critical to deepening our understanding of how SAA1-2 proteins contribute to specific pathophysiology *in vivo*. Such experiments could further reveal the conditions that critically affect the *in vivo* significance of SAA1-2. Furthermore, SAA1-2 proteins may be important in phenomena that were not investigated in the present study. For example, the inflammation status may have something to do with metabolism as we reported previously (30). The clarification of the *in vivo* functions of SAA1-2 in other such phenomena requires further examination.

In summary, we investigated the contribution of *Saa1-2* in liver inflammation caused by 4T1 breast cancers, finding that *Saa1-2* genes are dispensable for liver inflammation in this particular model. This study provides an example that the strong correlation in gene expression does not always reflect the *in vivo* significance and deepens our understanding of the relationship between systemic inflammation and acute phase response in cancer contexts.

## Materials and methods

### Mice

All animal experiment protocols were approved by the Animal Care and Use committee of Kyoto University. Mice were housed as described previously (20) in a 12-hour light/dark paradigm with food (CE-2, CLEA Japan, Inc., Tokyo, Japan) and water available *ad libitum*. Mice were randomly assigned to different experimental groups without any specific criterion. No blinding was performed. WT BALB/c mice were purchased from Japan SLC Inc. (Hamamatsu, Japan).

### Generation of KO mice

BALB/c *Saa1-2* KO mice were generated as described previously (20, 26). *In vitro* fertilized eggs stored were thawed and electroporated using CUY-EDIT II (BEX, Tokyo, Japan) (amplitude 20V, duration 3 msec., interval 97 msec. for twice) with two independent crRNAs (25 ng/µL: FASMAC, Kanagawa, Japan), tracrRNA (100 ng/µL: FASMAC), and purified recombinant Cas9 proteins (250 ng/µL: Thermo Fisher Scientific, MA, USA). The crRNA sequences are as follows:

*Saa1-2* crRNA1: 5′-CCACGUAUGAGGUGGCCCAUGGG-3′
*Saa1-2* crRNA2: 5′-CUGCAGCACACCCACGUAUGAGG-3′

Eggs at the 2-cell stage were transplanted into the oviduct of pseudopregnant mice. F0 mice were crossed with WT and F1 mice were obtained for generating KO mice (≥ F2).

### DNA extraction and genomic PCR

Genomic PCR to genotype mice was performed against DNAs prepared from the mouse tails. The tails were incubated with 90 µL of 50 mM NaOH (nacalai tesque, Kyoto, Japan) for more than 10 min at 95°C. 10 µL of 1 M Tris-HCl pH 8.0 (nacalai tesque) was then added to the reaction, followed by centrifugation at 10,000 × *g* for more than 10 min. The resulting supernatant was subjected to genomic PCR. Genomic PCR for genotyping was performed using KOD FX-Neo (TOYOBO, Osaka, Japan). The primers used in this experiment are shown in **Table S1**.

### Cell line and cancer transplantation

The 4T1 mouse breast cancer cell line (20) was cultured and maintained in RPMI1640 (nacalai tesque) in a 5% CO2 tissue culture incubator at 37°C as described previously (20). The media (RPMI1640) was supplemented with 10% fetal bovine serum (nacalai tesque) and 1% penicillin/streptomycin (nacalai tesque). The thawed cells were passaged once and then were transplanted to mice. 2.5×10^6^ 4T1 cells resuspended in 100 µL of RPMI1640 containing neither FBS nor penicillin/streptomycin were inoculated subcutaneously into the right flank of an 8–9-week-old BALB/c female mouse. In the sham-treated group, mice were given RPMI1640 supplemented with 10% FBS. Mice were sacrificed on day 14 post-transplantation and the liver and bone marrows were collected.

### RNA isolation, cDNA synthesis, and quantitative reverse transcription PCR

RNA isolation, cDNA synthesis, and RT-qPCR experiments were performed as described previously (20). Mouse livers were crushed in liquid nitrogen and homogenized with Trizol reagent (Thermo Fisher Scientific). Total RNAs were extracted from the homogenized supernatant using RNeasy Mini Kit (Qiagen, Venlo, Netherlands) according to the manufacturer’s instructions.

The bone marrows were collected essentially as described previously (31, 32). Briefly, the mouse right femurs from sham and 4T1-bearing mice were collected, cleaned of muscle tissue, and then were polished with gauze. The femurs were placed with the knee end down in a perforated 0.6-mL tube to which 80 μL of RNA*later* (Qiagen) was added and inserted in a 1.5 mL centrifuge tube, followed by centrifugation at 5,700 × *g* for 30 sec. The resulting pellets were suspended in 1 mL of Trizol reagent (Thermo Fisher Scientific) and proceeded with RNA extraction as described above.

Total RNAs (100 or 500 ng) were reverse-transcribed using Transcriptor First Strand cDNA synthesis kit (Roche, Basal, Switzerland) in a 10 μL reaction, which was then diluted 10-or 50-fold, respectively. qPCR experiments were performed using the StepOnePlus qPCR system (Applied Biosystems, CA, USA) and SYBR Green Master Mix (Roche). We used 2 μL of the obtained cDNA in a 10 μL qRT-PCR reaction. *Gapdh* was used as an internal control for the liver samples. *18S rRNA* was used as an internal control for the bone marrow samples.

### Western blotting

Crushed liver powders were lysed with lysis buffer (10 mM Tris-HCl pH8.0, 100 mM KCl, 2.5 mM MgCl_2_, 0.5% Triton Χ-100, cOmplete (Roche, Basel, Switzerland). The protein concentration was determined using the BCA protein assay kit (TaKaRa, Shiga, Japan) according to the manufacturer’s instructions. Twenty μg of the extracted protein were electrophoresed on a 15% sodium dodecyl sulfate (SDS) polyacrylamide gel for 1 hour at 150V and transferred to a polyvinylidene fluoride (PVDF) membrane (Millipore, MA, USA) for 1 hour at 72V. The membrane was incubated with 5% skim milk in Tris-buffered saline, 0.1% Tween20 (TBST) overnight at 4°C, and then was incubated with mouse serum amyloid A1/A2 antibody (1:1000 in Can Get Signal (TOYOBO, Osaka, Japan): AF2948, R&D Systems, MN, USA) for 2 hours at room temperature. The membrane was washed with TBST three times. Then, the membrane was incubated with goat IgG horseradish peroxidase-conjugated antibody (1:5000 in Can Get Signal (TOYOBO): HAF017, R&D systems) for 1 hour at room temperature. Following TBST-wash steps, signals were visualized using ECL Prime Western Blotting Detection reagent (Cytiva, Tokyo, Japan) and images were taken using Amersham ImageQuant800. The same protein samples were loaded onto a 15% SDS polyacrylamide gel and then stained with SYPRO Ruby Protein Gel Stain (Thermo Fisher Scientific) to confirm equal sample loading in each lane.

### Transcriptome analysis

Total RNAs were extracted as described above with RNase-Free DNase Set (Qiagen). RNA-seq libraries were generated using the NEBNext Globin&rRNA depletion kit and the NEBNext UltraII Directional RNA Library prep kit according to the manufacturer’s instructions (New England Biolabs, MA, USA). Sequencing experiments were performed using NextSeq 500 (Illumina; High Output Kit v2.5, 75 cycles). The obtained reads were filtered using fastp (version 0.20.1) (33) to remove low-quality sequences (< Q30), mapped to the mouse genome (version mm10) using Hisat2 (version 2.2.1) (34), and processed using Samtools (version 1.10) (35) and featureCounts (version 2.0.1) (36). Read counts were normalized with the reads per million per kilobase (RPKM) method. The generated gene expression matrix with RPKM scores is listed in **Table S2** and **Table S4**.

The generated gene expression matrix with RPKM scores was used to perform gene set enrichment analysis (GSEA) to interpret transcriptional profiles using GSEA 4.2.3 software (22, 37). Human hallmark collections were obtained from the Molecular Signatures Database (MSigDB, v7.5.1). A total of 1000 permutations were set to determine the significance of the enrichment for the gene sets, and the “Signal to noise” or “log_2_ ratio of classes” metric was conducted to rank the genes depending on sample size in each group according to the instruction. Enrichment score (ES), normalized enrichment score (NES), false discovery rate (FDR) and signatures were obtained. The enriched gene sets were defined as significant with FDR < 0.25. For differentially expressed gene (DEG) analyses, a read count matrix and a condition label vector were taken as input using the R package, edgeR (version 3.40.0) (38). First, we filtered out genes with very low expressions using CPM values rather than counts since they account for differences in sequencing depth between samples. Genes with CPM > 1 in at least 2 or more samples were taken into account. The trimmed mean of M values (TMM) method was applied to normalize the counts of retained genes among the different samples. The exact test function was performed to detect significantly expressed genes. The differentially expressed genes (DEGs) were defined as genes with |log2(fold change)| >1 and *p* value < 0.05. The volcano plots were depicted using ggplot2 to visualize DEGs (https://ggplot2.tidyverse.org/index.html). ImmuCellAI-mouse (24, 25), a bulk RNA-seq data deconvolution approach, was applied to estimate the abundance of 36 immune cell types using the default settings (**Table S3**).

### Flow cytometry

The livers of sham and 4T1-bearing mice were harvested on day 14 after transplantation. The obtained liver tissues were homogenized in 8 mL of RPMI1640 media containing 1.8 mg/mL Collagenase IV (WOR-CLS4-1, Worthington Biochemical Corporation, NJ, USA) and 112.3 µg/mL DNase I (11284932001, Roche). 8 mL of the suspension was filtered using a cell strainer (70 µm mesh) and then mixed with 4 mL of 90% Percoll solution (Sigma-Aldrich, MO, USA). The suspension was then centrifuged at 700 × *g* for 20 min. The red blood cells in the pellets were lysed with 1×Lysing buffer (Lysing Buffer 10× Concentrate (BD Biosciences) diluted by H_2_O (nacalai tesque)). The samples were washed with RPMI1640 media supplemented with 2% FBS. The obtained samples were stained for 15 min on ice in FACS buffer (2% FBS, 0.05% NaN_3,_ and 1×PBS) mixed with TruStain FcX™ (anti-mouse CD16/32) Antibody (1:200, Clone: 93, BioLegend, CA, USA) and eBioscience™ Fixable Viability Dye eFluor™ 780 (1:1000, Invitrogen, MA, USA). The samples were washed with 100 μL of FACS buffer. The washed samples were stained for 20 min on ice with Brilliant Violet 510™ anti-mouse CD45 Antibody (1:200, Clone: 30-F11, BioLegend), PE/Cyanine7 anti-mouse/human CD11b Antibody (1:200, Clone: M1/70, BioLegend), PE anti-mouse Ly-6C Antibody (1:200, Clone: HK1.4, BioLegend), and FITC anti-mouse Ly-6G Antibody (1:200, Clone: 1A8, BioLegend), and APC anti-mouse F4/80 Antibody (1:50, Clone: BM8, BioLegend) in FACS buffer. Following a wash step using FACS buffer, the stained samples were resuspended with FACS buffer and then filtered using a cell strainer (35 μm mesh) set in a 5 mL tube (Falcon). The resulting samples were analyzed using FACS Canto II (BD Bioscience, NJ, USA) and analyzed using FlowJo software (v10.801) (BD Biosciences).

### Statistics and data visualization

GraphPad Prism Software was used to analyze data. Data were displayed as mean ± SEM. Student’s *t* test was performed to analyze the statistical significance between groups unless otherwise indicated, and *p* value < 0.05 was considered statistically significant.

## Data availability statement

The data presented in the study are deposited in the DNA Data Bank of Japan (DDBJ) repository, accession number DRA014884 (https://www.ddbj.nig.ac.jp/index-e.html).

## Ethics statement

The animal study was reviewed and approved by the Animal Care and Use committee of Kyoto University.

## Author contributions

CH and RK performed experiments, analyzed data, constructed figures. AH, YN, RM, MY performed experiments. MT substantially contributed to the conception of this study. KK supervised the study. SK conceived and supervised the study, analyzed data, and wrote the paper. All authors contributed to the article and approved the submitted version.
