# Supplementary material for: Serum amyloid alpha 1-2 are not required for liver inflammation in the 4T1 murine breast cancer model
Source: Front Immunol. 2023 Feb 3;14:1097788. doi: 10.3389/fimmu.2023.1097788 (PMC9935569; doi:10.3389/fimmu.2023.1097788)
Supplement: Supplementary file 1 [file DataSheet_1.pdf]

Figure S1: The basic characteristics of *Saa1-2*

(A) A DNA sequence alignment of *Saa1* (accession number: CCDS21284.1) and *Saa2* (accession number: CCDS21285.1). The differences in their sequences are highlighted in red.

(B) The correlations of the mRNA abundances between *Saa1-2* and representative neutrophil related genes. *n* = 4.

A

```
      1         10        20        30        40        50        60        70
Saa1  ATGAAGCTAC TCACCAGCCT GGTCTTCTGC TCCCTGCTCC TGGGAGTCTG CCATGGAGGG TTTTTTTCAT
Saa2  ATGAAGCTAC TCACCAGCCT GGTCTTCTGC TCCCTGCTCC TGGGAGTCTG CCATGGAGGG TTTTTTTCAT

      71        80        90        100       110       120       130       140
Saa1  TTGTTCACGA GGCTTTCCAA GGGGCTGGGG ACATGTGGCG AGCCTACACT GACATGAAGG AAGCTAACTG
Saa2  TTATTGGGGA GGCTTTCCAA GGGGCTGGAG ACATGTGGCG AGCCTACACT GACATGAAGG AAGCTGGCTG

      141       150       160       170       180       190       200       210
Saa1  GAAAAACTCA GACAAATACT TCCATGCTCG GGGGAACTAT GATGCTGCTC AAAGGGGTCC CGGGGGAGTC
Saa2  GAAAGATGGA GACAAATACT TCCATGCTCG GGGGAACTAT GATGCTGCCC AAAGGGGTCC CGGGGGAGTC

      211       220       230       240       250       260       270       280
Saa1  TGGGCTGCTG AGAAAATCAG TGATGGAAGA GAGGCCTTTC AGGAATTCTT CGGCAGAGGA CATGAGGACA
Saa2  TGGGCTGCTG AGAAAATCAG TGATGCAAGA GAGAGCTTTC AGGAATTCTT CGGCAGAGGA CACGAGGACA

      281       290       300       310       320       330       340       350
Saa1  CCATTGCTGA CCAGGAAGCC AACAGACATG GCCGCAGTGG CAAAGACCCC AATTACTACA GACCTCCTGG
Saa2  CCATGGCTGA CCAGGAAGCC AACAGACATG GCCGCAGTGG CAAAGACCCC AATTACTACA GACCTCCTGG

      351       360
Saa1  ACTGCCTGAC AAATACTGA  369
Saa2  ACTGCCTGCC AAATACTGA  369
```

B

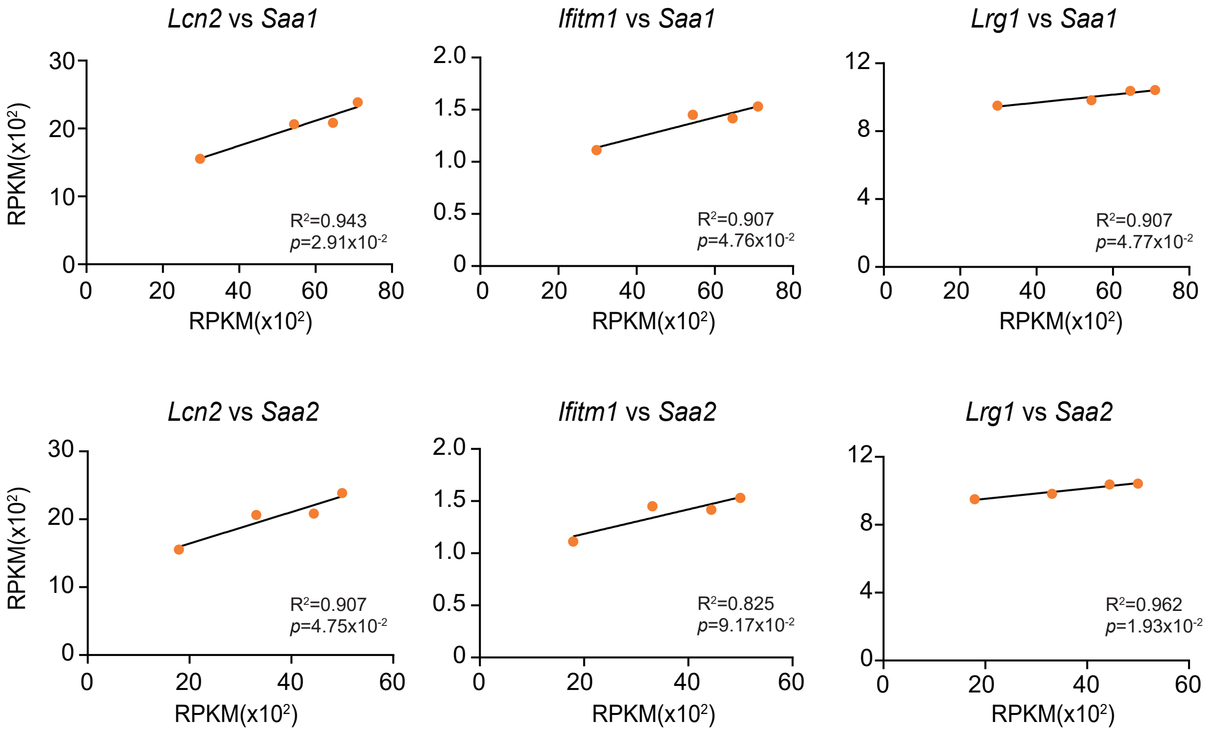

Figure S2: Western blot analysis of SAA1-2 proteins in the liver

Western blot analysis for SAA1-2 in the livers of sham and 4T1-bearing mice in WT and *Saa1-2* KO. Recombinant SAA1 protein (2948-SA: R&D systems, MN, USA) is included as a positive control.

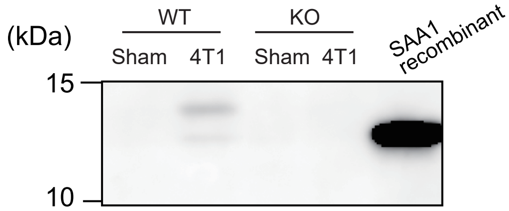

Figure S3: qPCR validations of the RNA-seq experiments in the liver

(A) This figure supports Fig. 3A. Top10 differentially expressed gene names are labelled.

(B) Venn diagrams showing the number of differentially expressed genes in the livers of 4T1-bearing WT and *Saa1-2* KO mice.

(C) qPCR analysis of *S100a8*, *Ly6g*, *Mpo*, and *Itgam* (*Cd11b*) in the livers of sham and 4T1-bearing mice in WT and *Saa1-2* KO (pooled from the four independent 4T1 transplantation experiments). Averaged fold change data normalized to the sham group in each genotype are presented as the mean ± SEM. *n.s.*, not significant, unpaired two-tailed Student's *t*-test. n = 11 for the sham groups, n = 13 for 4T1-bearing WT mice, and n = 14 for 4T1-bearing *Saa1-2* KO mice.

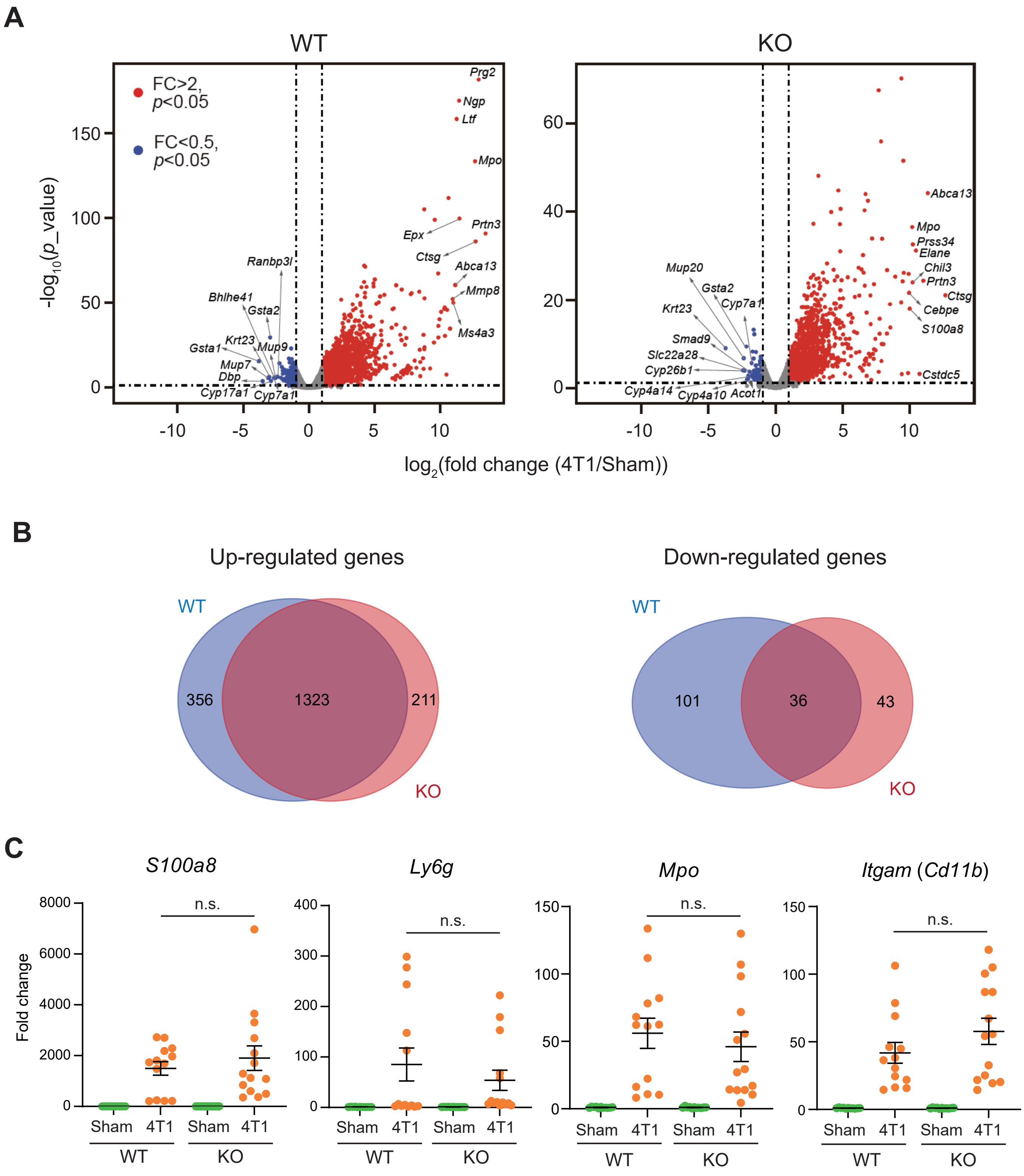

Figure S4: Gating strategies in the flow cytometry experiments

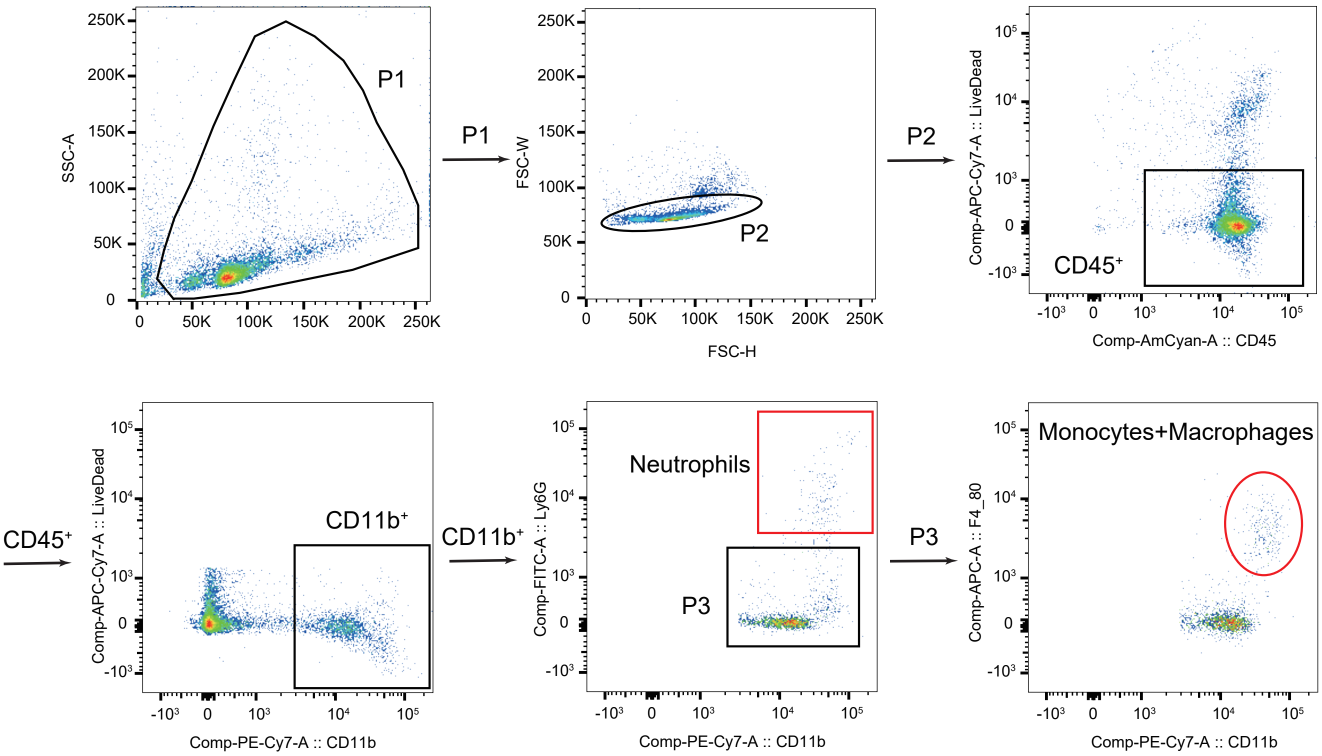

Figure S5: qPCR validations of the RNA-seq experiments in the bone marrow

(A) Venn diagrams showing the number of differentially expressed genes in the bone marrows of 4T1-bearing WT and *Saa1-2* KO mice.

(B) qPCR analysis of *Wfdc17*, *Lrg1*, *Ifitm1*, and *Saa3* in the bone marrows of sham and 4T1-bearing mice in WT and *Saa1-2* KO (pooled from the four independent 4T1 transplantation experiments).

Data are normalized with *18s rRNA* and are presented as the mean ± SEM. *n.s.*, not significant, unpaired two-tailed Student's *t*-test. *n* = 11 for sham-treated WT mice, *n* = 14 for 4T1-bearing WT mice, *n* = 9 for sham-treated WT mice, and *n* = 12 for 4T1-bearing *Saa1-2* KO mice.

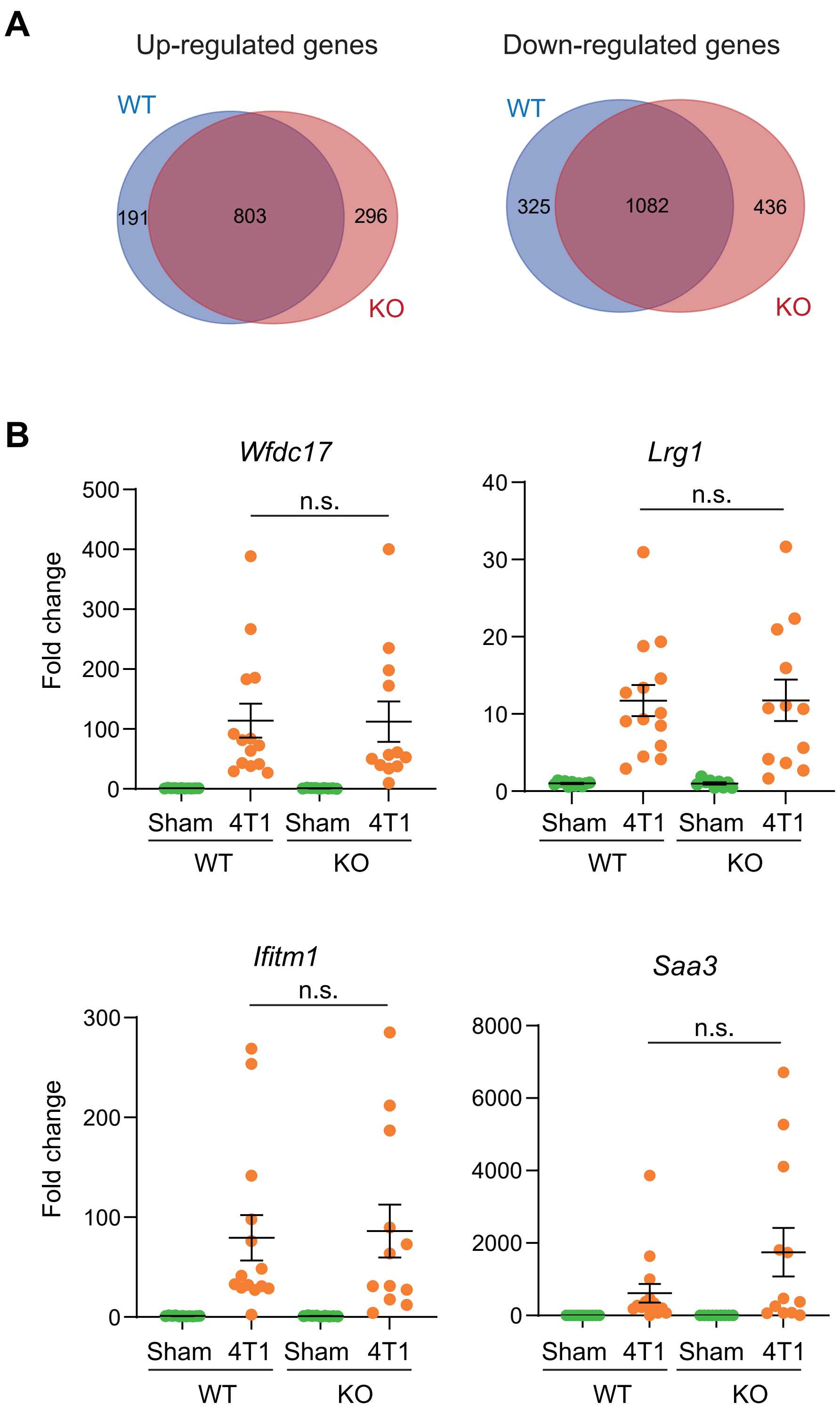

Figure S6: *Saa3* is induced in the livers of 4T1-bearing mice even in the absence of *Saa1-2*

(A) qPCR analysis of *Saa3* in the livers of sham and 4T1-bearing mice in WT and *Saa1-2* KO (pooled from the four independent 4T1 transplantation experiments). Averaged fold change data normalized to the sham group in each genotype are presented as the mean ± SEM. *n.s.*, not significant, unpaired two-tailed Student's *t*-test. n = 11 for the sham groups, n = 13 for 4T1-bearing WT mice, and n = 14 for 4T1-bearing *Saa1-2* KO mice.

(B) An amino acid sequence alignment of SAA1 (accession number: CCDS21284.1), SAA2 (accession number: CCDS21285.1) and SAA3 (accession number: CCDS21282.1).

A

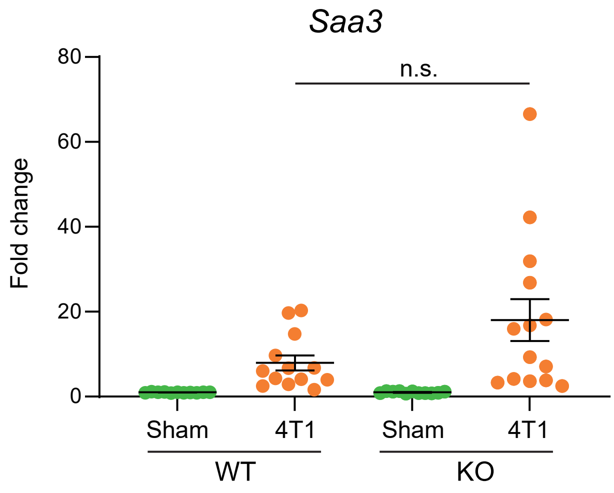

B

```
        1          10          20           30            40           50          60
SAA1   MKLLTSLVFC  SLLLGVCHGG   FFSFVHEAFQ   GAGDMWRAYT  DMKEANWKNS  DKYFHARGNY
SAA2   MKLLTSLVFC  SLLLGVCHGG   FFSF I GEAFQ  GAGDMWRAYT  DMKEAGWKDG  DKYFHARGNY
SAA3   MKPSI AI I LC  I L I LGVDSQR  WVQFMKEAGQ  GSRDMWRAYS  DMKKANWKNS  DKYFHARGNY

        61           70           80           90          100          110          120
SAA1   DAAQRGPGGV  WAAEKISDGR  EAFQEFFGRG  HEDTI ADQEA  NRHGRSGKDP  NYYRPPGLPD  KY
SAA2   DAAQRGPGGV  WAAEKISDAR  ESFQEFFGRG  HEDTMADQEA  NRHGRSGKDP  NYYRPPGLPA  KY
SAA3   DAARRGPGGA  WAAKVISDAR  EAVQKFTGHG  AEDSRADQFA  NEWGRSGKDP  NHFRPAGLPK  ZY
```
